# Supplementary figures and images for: Management of de novo metastatic hormone-sensitive prostate cancer: A comprehensive report of a single-center experience
Source: PLoS One. 2022 Aug 19;17(8):e0264800. doi: 10.1371/journal.pone.0264800 (PMC9390935; doi:10.1371/journal.pone.0264800)

## Slide 1
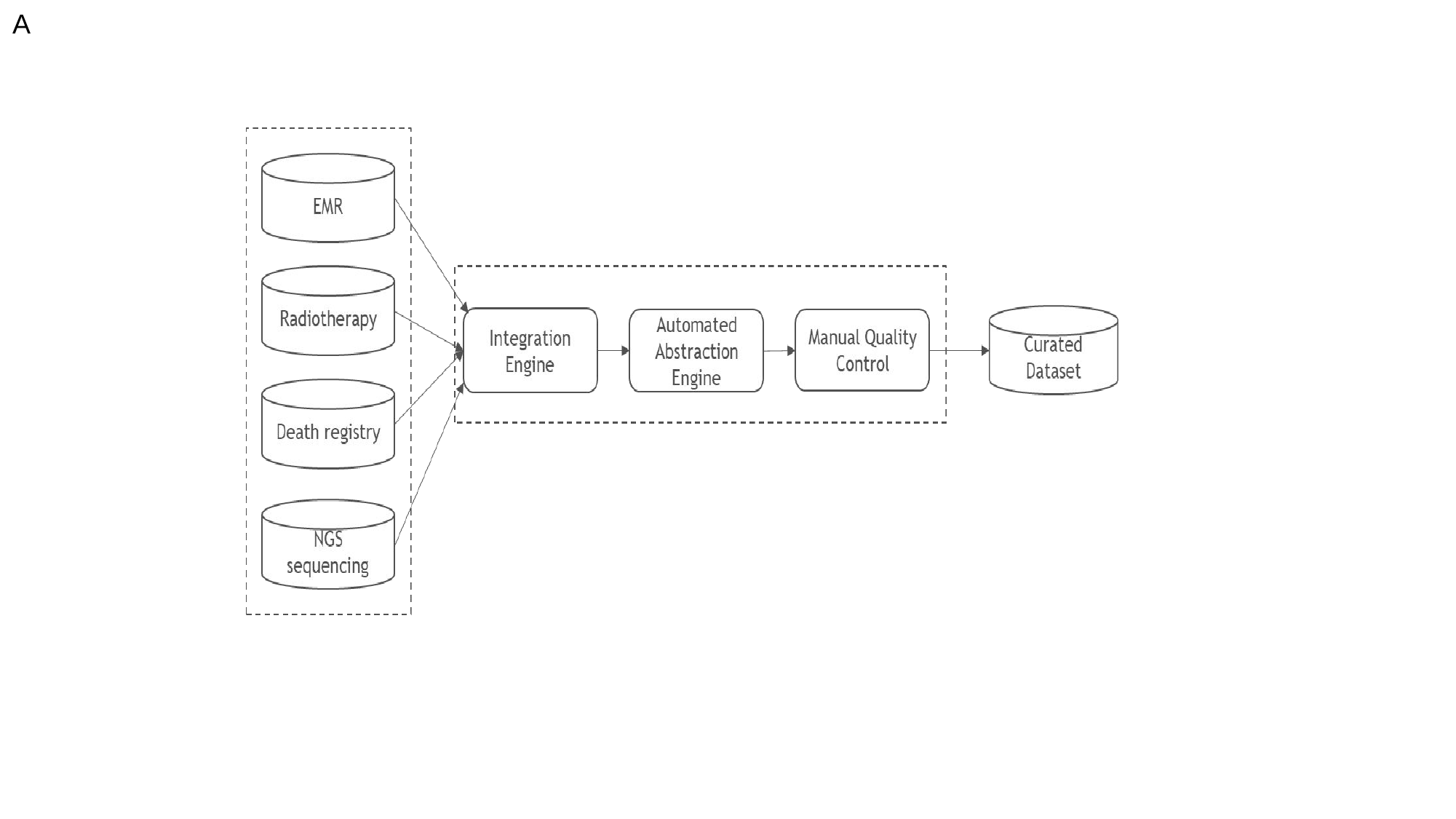

A

## Slide 2
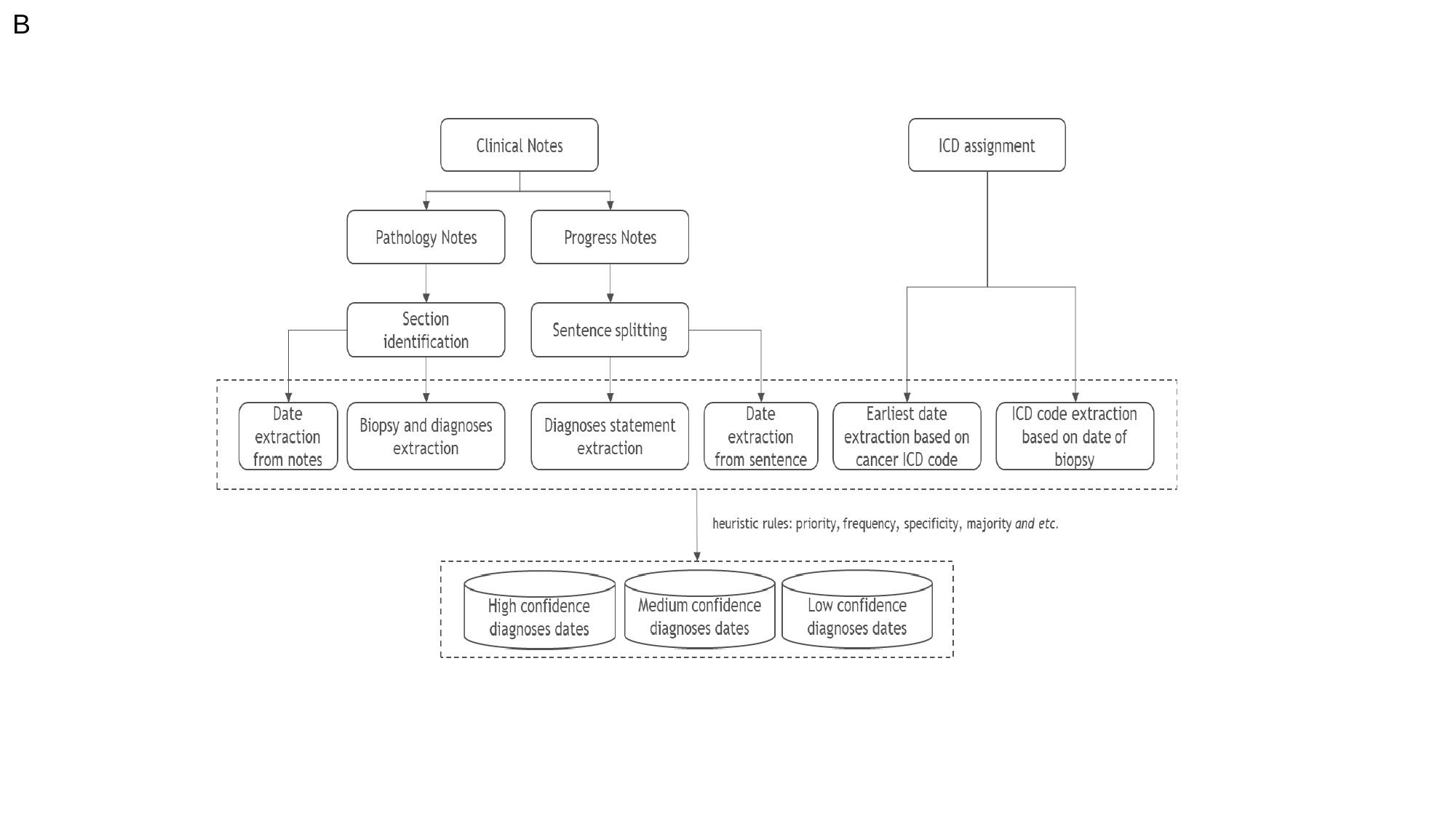

B

Supplement: S1 Fig — A. Sema4-automated oncology data retrieval and curation platform. B. Sema4 automated abstraction engine for cancer diagnosis. (PPTX) [file pone.0264800.s001.pptx]

Docetaxel NHA

Overall Survival

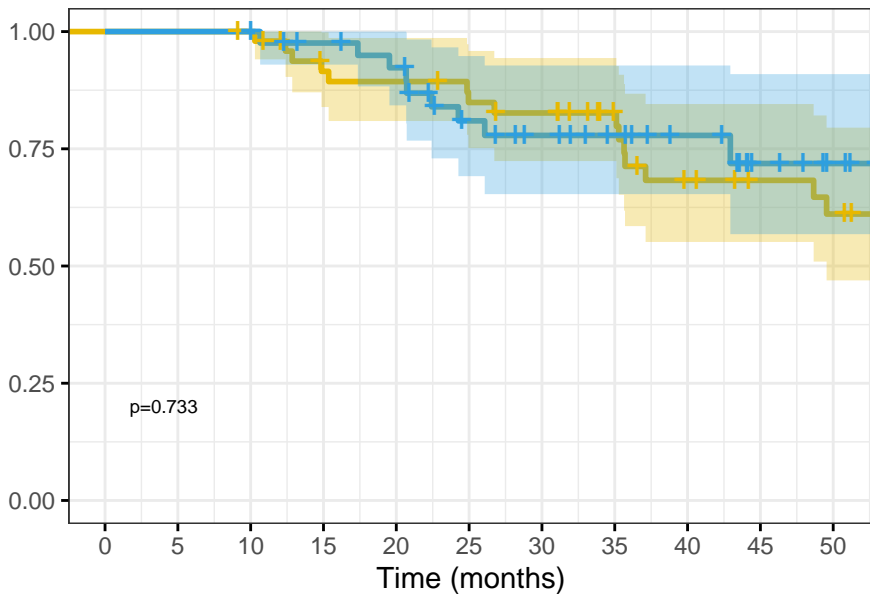

**Number at risk (number censored)**

|        |        |        |        |        |        |         |         |         |         |         |
|--------|--------|--------|--------|--------|--------|---------|---------|---------|---------|---------|
| 50 (2) | 50 (2) | 49 (3) | 42 (6) | 41 (6) | 38 (7) | 36 (8)  | 29 (15) | 22 (17) | 19 (20) | 17 (20) |
| 42 (0) | 42 (0) | 41 (1) | 38 (3) | 35 (4) | 26 (9) | 22 (12) | 18 (16) | 14 (20) | 7 (26)  | 3 (30)  |
| 0      | 5      | 10     | 15     | 20     | 25     | 30      | 35      | 40      | 45      | 50      |

Supplement: S3 Fig — (PDF) [file pone.0264800.s003.pdf]
