# Supplementary material for: Management of de novo metastatic hormone-sensitive prostate cancer: A comprehensive report of a single-center experience
Source: PLoS One. 2022 Aug 19;17(8):e0264800. doi: 10.1371/journal.pone.0264800 (PMC9390935; doi:10.1371/journal.pone.0264800)
Supplement: S2 Fig — (PPTX) [file pone.0264800.s002.pptx]

## Slide 1
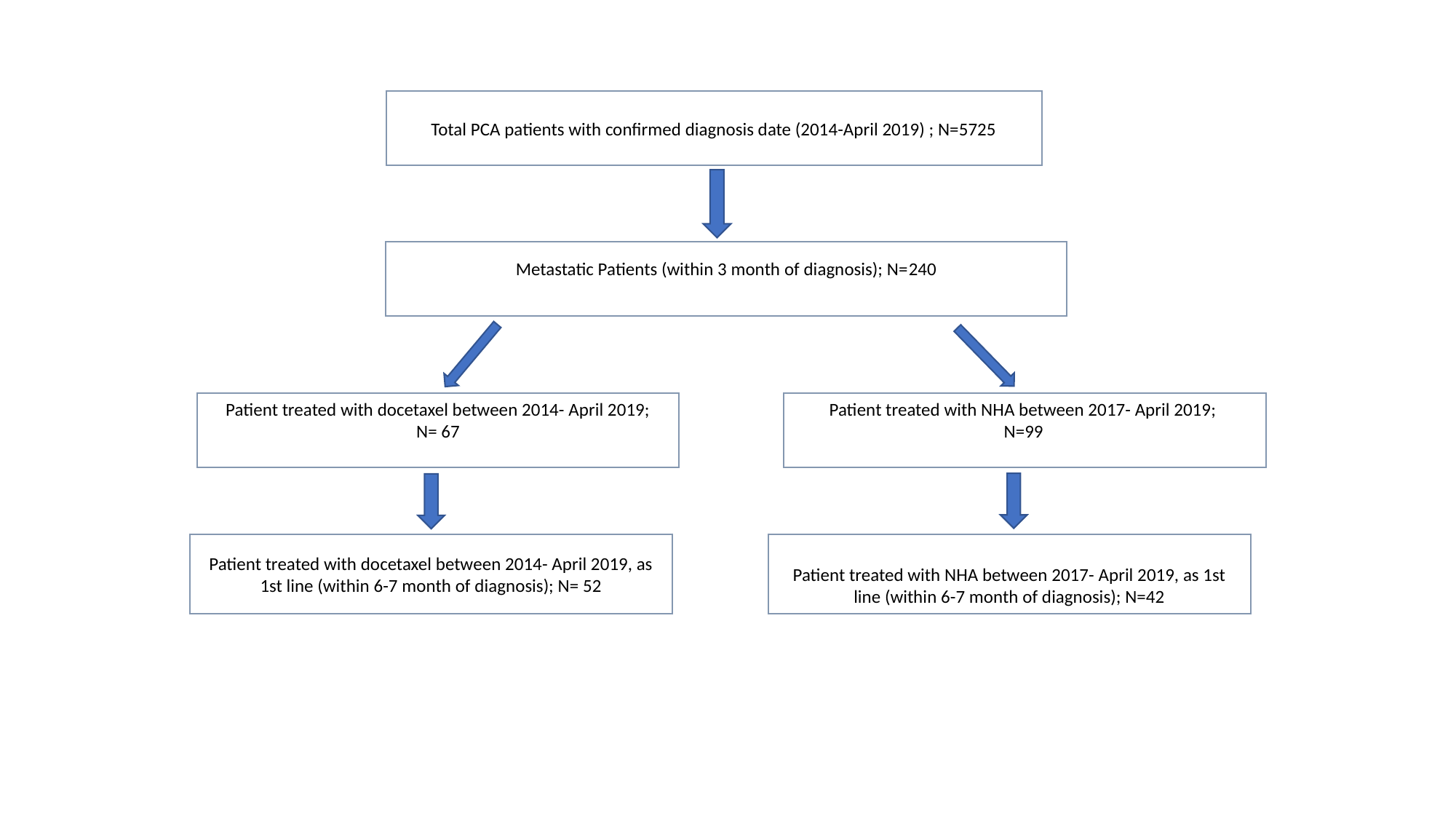

Total PCA patients with confirmed diagnosis date (2014-April 2019) ; N=5725
Metastatic Patients (within 3 month of diagnosis); N=240
Patient treated with NHA between 2017- April 2019;
                        N=99
Patient treated with docetaxel between 2014- April 2019; N= 67
Patient treated with docetaxel between 2014- April 2019, as 1st line (within 6-7 month of diagnosis); N= 52
Patient treated with NHA between 2017- April 2019, as 1st line (within 6-7 month of diagnosis); N=42
